# Investigation of Donor-Transmitted *Strongyloides stercoralis* Infections in Solid Organ Transplant Recipients, United States, 2012–2024

**DOI:** 10.3201/eid3207.260747

**Published:** 2026-07

**Authors:** Kerry R. Gainor, Dawn Blackburn, Pallavi Annambhotla, Sridhar V. Basavaraju, Katherine E. Bowden, Diana Martin, David A. Baran, Irene Frantzis, Irene D. Lytrivi, Julia Simkowski, Danielle Stanek, Susan P. Montgomery, Rebecca J. Chancey

**Affiliations:** Centers for Disease Control and Prevention, Atlanta, Georgia, USA (K.R. Gainor, D. Blackburn, P. Annambhotla, S.V. Basavaraiu, K.E. Bowden, D. Martin, S.P. Montgomery, R.J. Chancey); Cleveland Clinic Florida, Weston, Florida, USA (D.A. Baran); Columbia University Irving Medical Center, New York, New York, USA (I. Frantzis, I.D. Lytrivi); Cleveland Clinic, Cleveland, Ohio, USA (J. Simkowski); Florida Department of Health, Tallahassee, Florida, USA (D. Stanek).

**Keywords:** *Strongyloides stercoralis*, helminths, parasites, parasitic infection, organ donation, solid organ transplant, donor-derived infection, organ donor screening, United States

## Abstract

*Strongyloides stercoralis* is a parasitic nematode endemic in tropical and subtropical regions, including parts of the southeastern United States, that can be transmitted via organ donation. As of October 2025, the Organ Procurement and Transplant Network implemented new policy for screening in deceased US organ donors to reduce the risk for donor-derived *Strongyloides* infection. To assess the potential effect of policy changes, we reviewed investigations of suspected transplant-related strongyloidiasis in the United States conducted by the Centers for Disease Control and Prevention and partners for solid organ transplants occurring during 2012–2024. During that period, 21 proven donor-derived strongyloidiasis cases originated from 15 unscreened donors. Of donors who were screened, 31 seropositive donors resulted in ivermectin prophylaxis for 77 recipients, none of whom had disease develop. Our findings support the effectiveness of universal organ donor screening and prophylactic ivermectin treatment of recipients to prevent donor-derived *Strongyloides* infection.

Solid organ transplantations are lifesaving; however, they can pose major risks for illness and death from transplant-related complications, including infection with the parasite *Strongyloides stercoralis*. *S*. *stercoralis* is a parasitic nematode endemic in tropical and subtropical regions, including parts of the southeastern United States, and is estimated to affect 300–600 million persons worldwide (*1*–*3*). Risk factors for acquiring *Strongyloides* infection include living in or traveling to endemic regions, activities involving direct skin contact with soil, living in long-term care or correctional facilities, and human T-lymphotropic virus 1 (HTLV-1) infection (*1*,*4*,*5*).

*S*. *stercoralis* nematodes have a unique autoinfection cycle that enables them to establish asymptomatic, chronic infections even in immunocompetent persons (*1*,*4*–*6*). Immunocompromised persons, including solid organ recipients, have increased risk for severe disease because of infection reactivation or donor-derived infection (*6*,*7*). Severe disease is characterized by hyperinfection syndrome or disseminated *Strongyloides* infection, or both, and can have high mortality rates that reach up to 90% (*8*,*9*). Ivermectin is the treatment of choice for strongyloidiasis and has high efficacy, achieving cure rates of up to 96% in clinical trials (*6*,*10*–*13*).

To reduce the risks associated with donor-derived infections, the Organ Procurement and Transplant Network (OPTN) in the United States added *S. stercoralis* screening to policy 2.9, Required Deceased Donor Infectious Disease Testing, as part of the initiative to improve deceased donor evaluation for endemic diseases (*14*). This policy requires organ procurement organizations (OPO) to obtain serologic testing for *S. stercoralis* infection as part of the deceased donor evaluation process. The policy was approved in June 2023 and implemented in October 2025; testing all donors in the interim was highly recommended (*15*). When new donor testing information indicating a positive test becomes available, the OPO must notify the transplant centers. When a potential donor-derived disease transmission event is identified in a recipient, the transplant center must notify the OPO. The OPO then reports the suspected donor-derived infection case to OPTN in accordance with OPTN policy 15. Potential donor-derived transmission events reported to OPTN are reviewed by the ad hoc Disease Transmission Advisory Committee (DTAC) (*16*,*17*). The Centers for Disease Control and Prevention (CDC), as a member of DTAC, works with partners to routinely investigate pathogens of special interest, including *S. stercoralis*, as a public health response to ensure the ongoing safety of organ transplantation.

To assess the potential effect of implementing OPTN policy 2.9, we reviewed CDC-led investigations of transplant-related strongyloidiasis in the United States from 2012–2024. In addition, cases of confirmed donor-derived strongyloidiasis from 2023 and 2024 are described to emphasize the critical clinical features of *S. stercoralis* infection in organ recipients.

## Methods

### Data Compilation for Cases, 2012–2024

We extracted and reviewed records of suspected transplant-related strongyloidiasis cases previously collected by CDC during routine public health investigations for information related to transplant procedures and infection status of organ donor recipients. Data sources for descriptive analysis included case summary reports, donor and recipient demographic and clinical information as provided by OPOs and transplant hospitals, and laboratory test results collected during CDC-investigated cases of transplant-related strongyloidiasis. Donor information included age, sex, birthplace, exposure risks, *Strongyloides* serology testing results, and organs transplanted. Recipient information included time of symptom onset, time of diagnosis, testing results, exposure risks, and outcome at follow-up. We extracted the data by using a standardized Microsoft Excel (Microsoft, https://www.microsoft.com) data abstraction template and compiled the data in a database for descriptive analysis.

### Data Analysis

We reviewed the database of CDC-led case investigations from 2012–2024 to assess the potential effect of OPTN policy 2.9 by examining donor screening practices, donor risk characteristics, and recipient characteristics and outcomes to evaluate how universal donor screening could address gaps in identifying infected donors. We analyzed the data by using Microsoft Excel to generate frequency counts and proportions of donor and recipient variables. Variables analyzed include the year of transplant; donor age, sex, and birthplace; donor and recipient pretransplant and posttransplant testing results; the number of recipients and type of organ transplanted; and the recipient’s clinical course and outcome (Appendix Table 1). We used the clinical, laboratory, and epidemiologic data to assign a case determination to each organ recipient according to our case definitions (Figure; Appendix Table 2). We applied this approach to maintain consistency in classification. Case determinations were assigned by 1 independent reviewer and reassessed by 2 other independent reviewers by using predefined criteria (Figure; Appendix Table 2). We resolved discrepancies by consensus. The case definitions were proven, probable, possible, inconclusive, excluded, intervention without documented transmission, and no intervention no disease transmission (Appendix Table 2). We excluded investigations that did not have sufficient evidence of *S. stercoralis* infection in the donor and recipients from analysis. For analytical purposes, we classified donors as originating from countries considered endemic for *S. stercoralis* nematodes on the basis of CDC and the World Health Organization epidemiologic descriptions of regions with documented sustained transmission.

**Figure:**
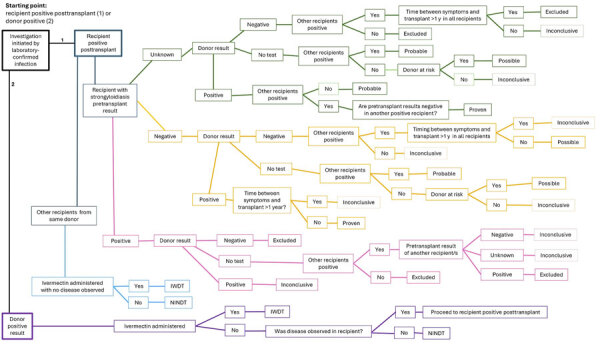
Case determination algorithm used for analysis in the Centers for Disease Control and Prevention investigations of potential donor-derived strongyloidiasis, United States, 2012–2024. Each investigation begins with a trigger, either recipient positive posttransplant (1) or donor positive (2), and follows the algorithm to a designated case determination based on clinical, laboratory, and epidemiologic data and our case definitions. Determination categories include proven, excluded, NINDT, IWDT, probable, possible, and inconclusive cases. IWDT, intervention without disease transmission; NINDT, no intervention and no disease transmission.

We describe investigations assigned as proven donor-derived infections in 2023 and 2024 for each donor and transplant-related strongyloidiasis recipient, corresponding to the period after policy approval and before full implementation across OPOs. Descriptions included donor and recipient data such as demographics, exposure risk factors, *S. stercoralis* and other diagnostic testing, treatment, and clinical course and outcomes that were previously collected as routine reporting for transplant procedures. This activity was reviewed by CDC, deemed not research, and was conducted consistent with applicable federal law and CDC policy (45 CFR part 46.102(l)(2), 21 CFR part 56; 42 USC §241(d); 5 USC §552a; 44 USC §3501 et seq.).

## Results

### Investigations from 2012–2024

During 2012–2024, CDC led 72 investigations of potential transplant-related strongyloidiasis involving 72 donors and 227 solid organ recipients. For this report, we excluded 8 investigations from analysis for lack of supportive evidence of *S. stercoralis* infection in the donor and recipients. The remaining 64 investigations available for analysis involved 64 donors (63 deceased and 1 living) and 197 recipients (Table; Appendix Table 1). Among the donors, 69% (n = 44) were male and 31% (n = 20) were female. Donors were 13–67 (median 46) years of age. Of donors, 53% (n = 34) were from countries other than the United States with documented endemic transmission of *S. stercoralis* nematodes, 14% (n = 9) of donors were born in the southeastern United States, 17% (n = 11) of donors were born in other parts of the mainland United States, 9% (n = 6) of donors were from Puerto Rico, and 6% (n = 4) of donors had an unknown country of origin.

**Table:** Summary of investigations of potential donor-derived strongyloidiasis, United States, 2012–2024*

Donor and recipient information	Total
Donors	64 (100)
Median age (range), y	46 (13–67)
Sex	
M	44 (69)
F	20 (31)
Birthplace	
International	34 (52)
United States	26 (41)
Unknown	4 (6)
Serologic testing	
Yes	60 (94)
No	4 (6)
Total organs transplanted	212
Recipients	197 (100)
Organs per recipient	
1	183 (93)
2	12 (6)
3	1 (0.5)
4	1 (0.5)
Recipients by organ types transplanted	
Right kidney	50 (25)
Left kidney	45 (23)
Liver	44 (22)
Heart	21 (11)
Lung, unspecified	7 (4)
Right lung	6 (3)
Left lung	6 (3)
Kidney, unspecified	4 (2)
Bilateral lung	4 (2)
Left kidney and pancreas	3 (1.5)
Liver and left kidney	2 (1)
Kidney and pancreas	1 (0.5)
Heart and left kidney	1 (0.5)
Heart and lung	1 (0.5)
Heart, liver, and left kidney	1 (0.5)
Liver, left kidney, pancreas, and bowel	1 (0.5)
Posttransplant strongyloidiasis diagnosis	
Positive	43 (22)
Negative	31 (16)
Unknown	123 (62)
Case determination	
Proven	22 (11)
Probable	3 (1.5)
Possible	1 (0.5)
Excluded	10 (5)
No intervention, no disease transmission	19 (10)
Intervention without disease transmission	125 (64)
Inconclusive	17 (9)
Outcome at follow-up	
Alive	167 (85)
Deceased	15 (8)
Unknown	15 (8)
Heart transplant recipients	
Recipients	24
Single-organ	21
Multi-organ	3
Case determination among heart transplant recipients	
Excluded, reactivation	2
Proven	7
Inconclusive	3
Intervention without disease transmission	10
No intervention, no disease transmission	1
Excluded, unknown	1

*Values are no. (%) except as indicated. Heart transplant recipients are presented separately for descriptive purposes and were not analyzed as a distinct risk group.

Specimens were submitted for serologic testing on 83% (n = 60) of the 72 donors. Of those, 53% (n = 32) were tested by the OPO referral laboratory at the time of organ procurement. Another 47% (n = 28) of specimens were tested by the OPO referral laboratory or CDC after transplant in response to reported recipient infection. For the remaining 4 of 64 investigations, donor specimens were not tested. Three of the 4 donor specimens were not tested because confirmatory serologic testing was unavailable at that time and because the samples did not meet the storage time requirements for testing at the OPO’s contracted laboratory. For donor specimen 4, the OPO determined the recipient’s infection was because of reactivation rather than donor-derived transmission and therefore did not test or send a sample to CDC for testing.

The investigations included 197 recipients who received solid organ transplants with a total of 212 organs transplanted (Table; Appendix Table 1). Among the recipients, 183 received a single organ and 14 received multiple organs during transplantation. The most frequently transplanted organs were the kidneys (55%, n = 108) and liver (24%, n = 48). A heart was transplanted in 24 patients (12% of total recipients), 21 as a single organ and 3 as part of a multiorgan transplant. Of the heart transplant recipients, 7 acquired donor-derived strongyloidiasis and 2 tested seropositive for *S. stercoralis* infection on serum samples collected before transplant, consistent with pretransplant infection, and subsequently experienced reactivation posttransplant. The remaining heart recipient outcomes are provided (Table). Less frequently transplanted organs include lungs and other multiorgan combinations.

On the basis of the case determination algorithm (Figure), 11% (n = 22) of cases met the definition of proven transmission, characterized by confirmed *S. stercoralis* infection in both the donor and >1 recipient, with documented absence of pretransplant infection in the recipient. Three (2%) cases were classified as probable transmission because evidence strongly suggested donor origin but not all criteria for proven transmission were fulfilled. Possible transmission was identified in 1 (0.5%) case; transmission was suspected but the available evidence was insufficient to meet the criteria for proven or probable. Among the 26 proven, probable, and possible cases, the most frequently transplanted organ was the kidney (46%, n = 12 cases), followed by the heart (27%, n = 7 cases). We excluded 10 (5%) cases on the basis of evidence indicating the recipient had reactivated chronic infection acquired before transplant or new infection acquired posttransplant. The organs most frequently transplanted in the excluded cases were the lungs (40%, n = 4 cases). In 63% (n = 125) of cases, recipients received treatment posttransplant and had no observed transmission; we categorized those cases as intervention without disease transmission. In 9% (n = 17) of cases, we could not determine the strongyloidiasis cause or we assigned the case to another classification; we designated those cases as inconclusive. Of the inconclusive cases, kidneys (65%, n = 11 cases) were the most frequently transplanted organs. The remaining 10% (n = 19) of cases received no prophylaxis and no transmission was documented; we assigned those cases no intervention no disease transmission status. Complications related to strongyloidiasis resulted in the death of 5% (n = 10) of recipients.

In the proven and probable investigations, 15 donors were from non-US countries and 4 donors were from the United States and Puerto Rico. Among the 25 affected recipients, 32% (n = 8) died because of complications related to strongyloidiasis. Since the approval of policy 2.9 in 2023, there were 5 proven cases and 1 probable case of donor-derived infections involving 5 unscreened donors.

### Proven Donor-Derived Public Health Investigations, 2023–2024

The following select, proven investigations occurred after June 26, 2023, when OPTN approved policy 2.9, emphasizing ongoing gaps in screening protocols and the need to implement universal donor screening across all OPOs to prevent future donor-derived strongyloidiasis. Transplant case identifiers correspond to those listed in Appendix Table 1.

#### Transplant Case Identification 2023-3

The donor was a 33-year-old man born in Guatemala. The OPO performed *S. stercoralis* serology at the time of death, and results were reported positive 3 days later. The transplant centers of all 4 recipients were notified. Of the 4 recipients, the liver, right kidney, and left kidney recipients received prophylactic ivermectin; none reported disease development. The heart recipient did not receive prophylaxis. At 11 weeks posttransplant, the heart recipient reported a 2-week history of decreased appetite, abdominal pain, bloating, and diarrhea and was hospitalized. Fecal ova and parasite examination was positive for *S. stercoralis* infection. Upon receipt of the positive result, the heart recipient was administered ivermectin. Despite medical intervention, the recipient’s condition declined, leading to death at 87 days posttransplant. The recipient’s pretransplant serology was negative for *S. stercoralis* infection, supporting the absence of detectable infection before transplantation. The recipient was born in China and arrived in the United States 4 years before the heart transplant. Although the donor screening result was communicated 1 day posttransplant, the message did not reach the appropriate members of the heart transplant team, and prophylactic ivermectin was not administered. Corrective measures have been established to improve communication and follow-up in the future.

#### Transplant Case Identification 2024-1

The donor was a 52-year-old woman originally from Guatemala. She traveled to Guatemala most recently in 2022 and had no other travel history. She was not tested for *S. stercoralis* infection before the time of organ transplant in recipients. A serum sample from the donor was submitted for testing after the heart and left kidney recipients’ diagnosis of strongyloidiasis. Results of *S. stercoralis* IgG ELISA testing were positive at both the OPO referral laboratory and CDC. Organs transplanted were heart, right and left kidneys, lung, and liver.

The heart recipient was born in OPTN region 1 (Northeast United States, New England region) and resided in region 10 (Midwest United States, Great Lakes region) at the time of transplant. The recipient traveled to a state in region 11 (Southeast United States, southern Atlantic region) and had no other recent travel or exposure risks. The result of a *S. stercoralis* antibody test in the heart recipient was negative pretransplant. On day 62 posttransplant, the heart recipient complained of worsening pain, nausea, and diarrhea. Intestinal biopsy on day 69 posttransplant and skin biopsies on day 75 posttransplant revealed acute duodenitis with *S. stercoralis* organisms and cutaneous strongyloidiasis. Serology and fecal ova and parasite microscopic examination were positive for *S. stercoralis* infection on day 75 posttransplant. Bronchoscopy with bronchoalveolar lavage (BAL) demonstrated diffuse alveolar hemorrhage, and *Strongyloides* larvae were observed in BAL cultures. The recipient was treated for disseminated strongyloidiasis with oral ivermectin before transitioning to subcutaneous ivermectin. Other conditions included vancomycin-resistant *Enterococcus* bacteremia, pneumonia, and suspected meningitis. As of 89 days posttransplant, the recipient remained in the intensive care unit requiring ventilator support. Abnormal mental status, encephalopathy, and delirium were observed. At follow-up 118 days posttransplant, the recipient continued to be hospitalized and was slowly improving.

The left kidney recipient was a resident of OPTN region 10, had no recent travel history and had pretransplant serology negative for *Strongyloides* antibodies. On day 67 posttransplant, the recipient reported complaints of intractable vomiting and nausea. *Strongyloides* larvae were observed in sputum cultures and fecal samples were positive for *S. stercoralis* larvae. The recipient was treated with oral albendazole for 27 days and oral ivermectin for 2 days, then subcutaneous ivermectin for 25 days. Complications experienced by the recipient included respiratory failure, septic shock, encephalopathy, bacteremia, and fluid accumulation around the transplanted kidney. At follow-up 97 days posttransplant, the recipient remained in the ICU.

Pretransplant and posttransplant serum samples from the right kidney and lung recipients were not tested, and both recipients were given ivermectin. The liver recipient’s pretransplant and posttransplant serology were negative, and the recipient was also treated with ivermectin.

#### Transplant Case Identification 2024-2

The donor was a 33-year-old Hispanic man who was born in and resided in Honduras until 17 years of age, when he moved to the United States. Prior to organ donation, he was not screened for *S. stercoralis* infection. After notification of strongyloidiasis in the heart recipient, the archived donor serum was tested by the OPO referral laboratory with a positive result for *Strongyloides* antibodies and subsequently tested negative at CDC. This discordance is likely attributable to differences in assay methodologies, antigens used, and assay performance. Donor organs transplanted include heart, right and left kidneys, and liver.

The heart recipient was from OPTN region 9 (Northeast United Sates, including New York and western Vermont), did not have international travel or exposure risk for *S. stercoralis* infection, and had serum samples that were negative when tested pretransplant. At 161 days posttransplant, the heart recipient reported complaints of abdominal pain, nausea, and weight loss. Clinical course was complicated by diffuse pulmonary disease characterized by extensive nodular and reticulonodular opacities, pulmonary hemorrhage, and multi–organ system involvement, including renal failure and anemia requiring blood transfusions. The recipient was treated with antimicrobial drugs and steroids for possible *Pneumocystis* pneumonia. A commercial metagenomic molecular test identified *S. stercoralis* DNA in plasma, which was confirmed by identification of *Strongyloides* larvae in a BAL sample. Treatment with oral ivermectin and albendazole was initiated 171 days posttransplant, and the patient was transitioned to subcutaneous ivermectin. After initiation of ivermectin therapy, the heart recipient experienced cerebral edema and poor neurologic function and was placed on veno-venous extracorporeal membrane oxygenation and continuous renal replacement therapy because of worsening clinical status. At 274 days posttransplant, the recipient had a tracheostomy, remained on continuous renal replacement therapy, regained consciousness, and could communicate. 

Pretransplant serum from the left and right kidney recipients could not be tested, and posttransplant serology was negative. Both recipients received ivermectin. The liver recipient died from causes unrelated to strongyloidiasis and could not be tested.

## Discussion

Donor-derived infections are a rare but serious complication of solid organ transplantation (*17*–*19*). Parasitic infections, particularly with *S*. *stercoralis*, can be fatal in recipients, often because of delayed diagnosis (*2*,*8*,*17*). To reduce risk, professional society guidelines have recommended targeted screening of deceased donors on the basis of epidemiologic risk factors such as country of origin (*2*,*8*,*20*,*21*). However, adherence to screening protocols for *S. stercoralis* infection in organ donors remained suboptimal. In 2016, only 10% of OPOs reported screening donors on the basis of risk factors, and by 2019, only 24% had adopted either targeted or universal screening protocols (*22*,*23*). In the cases investigated (Appendix Table 1), 50% donors were not screened before transplant. Of those, 94% had epidemiologic risk factors, and 40% of donors transmitted *S. stercoralis* infection to 18 recipients, leading to 3 strongyloidiasis-related deaths.

More recently, the OPTN added universal *S*. *stercoralis* screening to policy 2.9 (*14*). Implementing universal screening, which involves testing all donors regardless of epidemiologic risk, could reduce variability in the application of screening guidelines. Testing all donors would also address the limitations of targeted screening, which might miss donors with unrecognized risk, and help prevent severe disease that is difficult to treat. In the time between policy 2.9 approval and implementation, donor-derived *Strongyloides* infections continued to occur, with 2 investigations involving donors from endemic regions who were not screened at the time of organ procurement. In both cases, multiple recipients had serious complications of hyperinfection and disseminated disease, including respiratory failure, sepsis, encephalopathy, and prolonged ICU stays.

In our investigations, 9 donors and 4 recipients were positive for *S*. *stercoralis* infection and lacked international travel history, suggesting *S. stercoralis* infection was acquired in the United States (Appendix Table 1). *S*. *stercoralis* nematodes have been documented in parts of the Appalachian region since the 1940s and more recently in several other US states (*24*–*31*). However, *S*. *stercoralis* infection is not nationally notifiable or reportable in any state, and current comprehensive national data remain scarce (*31*,*32*). Therefore, the true prevalence and geographic distribution are unknown, complicating accurate risk assessment and limiting the feasibility of targeted screening strategies. The 9 donors described here lived in US regions historically found to have *Strongyloides* transmission or had identifiable risk factors such as incarceration, communal living, or occupational exposure, and some had history of both US region residence and other risk factors. Donor histories might have been incomplete and lacked documentation of exposure risk factors and therefore donor screening was not undertaken (*19*,*33*). Screening all donors, even those with unrecognized risks, would help prevent donor-derived infection and identify potential donors who might have acquired *S. stercoralis* infection in the United States.

Screening for *S. stercoralis* infection enables early identification of seropositive donors. Organs from seropositive donors have been successfully transplanted without adverse outcomes when recipients received prophylactic treatment (*19*,*34*–*36*). Although serologic tests might produce false positives in a low prevalence population, this should not preclude safe organ use (*2*,*37*). Treating recipients with ivermectin is a well-tolerated and effective low-risk intervention (*6*,*15*,*38*,*39*). Our assessment identified 31 of 32 screened donors who were seropositive, prompting prophylactic ivermectin treatment in 77 recipients. No disease was reported in those recipients at follow-up. The success of universal screening is contingent on timely and accurate communication of positive results. In 3 investigations with a positive donor, communication errors led to 4 recipients not receiving prophylactic ivermectin, resulting in deaths attributable to strongyloidiasis. In contrast, other recipients from the same donors were treated after donor screening and remained disease-free, suggesting that screening might have prevented donor-derived strongyloidiasis in these recipients. The recipient outcomes observed emphasize the importance of donor screening to identify seropositive donors, timely effective communication of results, and early ivermectin treatment.

The first limitation of our study is that the retrospective design of the analysis is limited by the availability and accuracy of historical records. Donor and recipient risk factors, travel histories, and testing results might have been incompletely documented. Those factors might have affected the ability to definitively assign appropriate case determination. Second, the scope of this report is limited to CDC-led investigations and might not represent all donor-derived strongyloidiasis cases in the United States from 2012–2024. Third, serology methods used for donor screening, although valuable, have inherent limitations. Sensitivity and specificity of commonly used serologic tests can vary by assay and reference standard. Reported sensitivities range approximately from 70% to 95%, whereas specificities are generally high, ranging from ≈90% to 99% (*40*–*42*). False positives can occur in low prevalence populations or from cross reactivity with other nematodes, whereas false negatives are possible in immunocompromised persons and hemodiluted samples. Last, our analysis primarily includes investigations conducted before the approval of policy 2.9, which limits our ability to determine the direct measurable effect of the policy on donor screening practices and recipient outcomes; however, the 2 cases described from 2023 and 2024 highlight the importance of screening donors.

In conclusion, universal screening of all donors, regardless of risk, is expected to reduce donor-derived strongyloidiasis in recipients by providing early detection, timely preventative intervention, and help reduce severe disease that is difficult to treat. Future comparative analyses should be performed to evaluate the effect of *S. stercoralis* screening implementation across OPOs prepolicy and postpolicy implementation and associated recipient outcomes. Our analysis confirms support of universal *S. stercoralis* screening of US solid organ donors. 

Appendix Additional information about investigation of donor-transmitted *Strongyloides stercoralis* infections in solid organ transplant recipients, United States, 2012–2024.

## References

[1] Lo NC, Addiss DG, Buonfrate D, Amor A, Anegagrie M, Bisoffi Z, et al. Review of the WHO guideline on preventive chemotherapy for public health control of strongyloidiasis. Lancet Infect Dis. 2025;25:e146–52. 10.1016/S1473-3099(24)00595-4 39481419 PMC11871984

[2] La Hoz RM, Morris MI, Infectious Diseases AST, Infectious Diseases AST. Community of Practice. Intestinal parasites including *Cryptosporidium, Cyclospora, Giardia*, and *Microsporidia, Entamoeba histolytica, Strongyloides, Schistosomiasis*, and *Echinococcus*: guidelines from the American Society of Transplantation Infectious Diseases Community of Practice. Clin Transplant. 2019;33:e13618. 10.1111/ctr.13618 31145496

[3] Buonfrate D, Bisanzio D, Giorli G, Odermatt P, Fürst T, Greenaway C, et al. The global prevalence of *Strongyloides stercoralis* infection. Pathogens. 2020;9:468. 10.3390/pathogens9060468 32545787 PMC7349647

[4] Nutman TB. Human infection with *Strongyloides stercoralis* and other related *Strongyloides* species. Parasitology. 2017;144:263–73. 10.1017/S0031182016000834 27181117 PMC5563389

[5] Yeh MY, Aggarwal S, Carrig M, Azeem A, Nguyen A, Devries S, et al. *Strongyloides stercoralis* infection in humans: a narrative review of the most neglected parasitic disease. Cureus. 2023;15:e46908. 10.7759/cureus.46908 37954715 PMC10639005

[6] Keiser PB, Nutman TB. *Strongyloides stercoralis* in the immunocompromised population. Clin Microbiol Rev. 2004;17:208–17. 10.1128/CMR.17.1.208-217.2004 14726461 PMC321465

[7] Roseman DA, Kabbani D, Kwah J, Bird D, Ingalls R, Gautam A, et al. *Strongyloides stercoralis* transmission by kidney transplantation in two recipients from a common donor. Am J Transplant. 2013;13:2483–6. 10.1111/ajt.12390 23919410 PMC3785548

[8] Kim JH, Kim DS, Yoon YK, Sohn JW, Kim MJ. Donor-derived strongyloidiasis infection in solid organ transplant recipients: a review and pooled analysis. Transplant Proc. 2016;48:2442–9. 10.1016/j.transproceed.2015.11.045 27742318

[9] Mobley CM, Dhala A, Ghobrial RM. *Strongyloides stercoralis* in solid organ transplantation: early diagnosis gets the worm. Curr Opin Organ Transplant. 2017;22:336–44. 10.1097/MOT.0000000000000428 28562417

[10] Henriquez-Camacho C, Gotuzzo E, Echevarria J, White AC Jr, Terashima A, Samalvides F, et al. Ivermectin versus albendazole or thiabendazole for *Strongyloides stercoralis* infection. Cochrane Database Syst Rev. 2016;2016:CD007745. 10.1002/14651858.CD007745.pub3 26778150 PMC4916931

[11] Gann PH, Neva FA, Gam AA. A randomized trial of single- and two-dose ivermectin versus thiabendazole for treatment of strongyloidiasis. J Infect Dis. 1994;169:1076–9. 10.1093/infdis/169.5.1076 8169394

[12] Datry A, Hilmarsdottir I, Mayorga-Sagastume R, Lyagoubi M, Gaxotte P, Biligui S, et al. Treatment of *Strongyloides stercoralis* infection with ivermectin compared with albendazole: results of an open study of 60 cases. Trans R Soc Trop Med Hyg. 1994;88:344–5. 10.1016/0035-9203(94)90110-4 7974685

[13] Marti H, Haji HJ, Savioli L, et al. A comparative trial of a single-dose ivermectin versus three days of albendazole for treatment of *Strongyloides stercoralis* and other soil-transmitted helminths. Am J Trop Med Hyg. 1996;55:477–81. 10.4269/ajtmh.1996.55.477 8940976

[14] Organ Procurement and Transplantation Network. Policy 2.9: improve deceased donor evaluation for endemic diseases. 2023 [cited 2025 Mar 12]. https://optn.transplant.hrsa.gov/media/lrxfreqj/dtac_endemics_policy-notice_june23bod.pdf

[15] Ad Hoc Disease Transmission Advisory (DTAC). Recognizing seasonal and geographically endemic infections in organ donors: considerations during deceased and living donor evaluation. 2024 [cited 2025 May 29]. https://optn.transplant.hrsa.gov/media/q4xfi14u/policy_notice_dtac_endemic_guidance.pdf

[16] Organ Procurement and Transplantation Network. Policy 15: identification of transmissible diseases. [cited 2025 Jul 15]. https://optn.transplant.hrsa.gov/media/eavh5bf3/optn_policies.pdf

[17] Kaul DR, Vece G, Blumberg E, La Hoz RM, Ison MG, Green M, et al. Ten years of donor-derived disease: a report of the disease transmission advisory committee. Am J Transplant. 2021;21:689–702. 10.1111/ajt.16178 32627325

[18] Ison MG, Nalesnik MA. An update on donor-derived disease transmission in organ transplantation. Am J Transplant. 2011;11:1123–30. 10.1111/j.1600-6143.2011.03493.x 21443676

[19] Hogan JI, Mehta SA. *Strongyloides stercoralis* infection in solid organ transplant recipients. Curr Opin Infect Dis. 2024;37:367–75. 10.1097/QCO.0000000000001046 39082077

[20] La Hoz RM. Minimizing the risk of donor-derived events and maximizing organ utilization through education and policy development. Infect Dis Clin North Am. 2023;37:443–58. 10.1016/j.idc.2023.05.002 37302913

[21] Schwartz BS, Mawhorter SD, Infectious Diseases AST, Infectious Diseases AST. Community of Practice. Parasitic infections in solid organ transplantation. Am J Transplant. 2013;13(Suppl 4):280–303. 10.1111/ajt.12120 23465021

[22] Abanyie FA, Valice E, Delli Carpini KW, Gray EB, McAuliffe I, Chin-Hong PV, et al. Organ donor screening practices for *Strongyloides stercoralis* infection among US organ procurement organizations. Transpl Infect Dis. 2018;20:e12865. 10.1111/tid.12865 29512242 PMC6190834

[23] Theodoropoulos NM, Greenwald MA, Chin-Hong P, Ison MG. Testing deceased organ donors for infections: an organ procurement organization survey. Am J Transplant. 2021;21:1924–30. 10.1111/ajt.16552 33621430

[24] Siddiqui AA, Berk SL. Diagnosis of *Strongyloides stercoralis* infection. Clin Infect Dis. 2001;33:1040–7. 10.1086/322707 11528578

[25] Starr MC, Montgomery SP. Soil-transmitted helminthiasis in the United States: a systematic review—1940–2010. Am J Trop Med Hyg. 2011;85:680–4. 10.4269/ajtmh.2011.11-0214 21976572 PMC3183777

[26] Singer R, Sarkar S. Modeling strongyloidiasis risk in the United States. Int J Infect Dis. 2020;100:366–72. 10.1016/j.ijid.2020.09.002 32896663

[27] Croker C, She R. Increase in reports of *Strongyloides* infection—Los Angeles County, 2013–2014. MMWR Morb Mortal Wkly Rep. 2015;64:922–3. 10.15585/mmwr.mm6433a8 26313477

[28] Leapley A, Cruze A, Mejia-Echeverry A, et al.; Centers for Disease Control and Prevention. Notes from the field: *Strongyloides* infection among patients at a long-term care facility—Florida, 2010–2012. MMWR Morb Mortal Wkly Rep. 2013;62:844. 24153317 PMC4585618

[29] Berk SL, Verghese A, Alvarez S, Hall K, Smith B. Clinical and epidemiologic features of strongyloidiasis. A prospective study in rural Tennessee. Arch Intern Med. 1987;147:1257–61. 10.1001/archinte.1987.00370070071011 3606282

[30] Jones JM, Hill C, Briggs G, Gray E, Handali S, McAuliffe I, et al. Notes from the field: strongyloidiasis at a long-term–care facility for the developmentally disabled—Arizona, 2015. MMWR Morb Mortal Wkly Rep. 2016;65:608–9. 10.15585/mmwr.mm6523a5 27310213

[31] Singer R, Xu TH, Herrera LNS, Villar MJ, Faust KM, Hotez PJ, et al. Prevalence of intestinal parasites in a low-income Texas community. Am J Trop Med Hyg. 2020;102:1386–95. 10.4269/ajtmh.19-0915 32207401 PMC7253135

[32] Davis S, Bosserman E, Montgomery S, Woodhall D, Russell ES; Centers for Disease Control and Prevention. Notes from the field: Strongyloidiasis in a rural setting—southeastern Kentucky, 2013. MMWR Morb Mortal Wkly Rep. 2013;62:843. 24153316 PMC4585617

[33] White SL, Rawlinson W, Boan P, Sheppeard V, Wong G, Waller K, et al. Infectious disease transmission in solid organ transplantation: donor evaluation, recipient risk, and outcomes of transmission. Transplant Direct. 2018;5:e416. 10.1097/TXD.0000000000000852 30656214 PMC6324914

[34] Abanyie FA, Gray EB, Delli Carpini KW, Yanofsky A, McAuliffe I, Rana M, et al. Donor-derived *Strongyloides stercoralis* infection in solid organ transplant recipients in the United States, 2009–2013. Am J Transplant. 2015;15:1369–75. 10.1111/ajt.13137 25703251 PMC4747246

[35] Multani A, Deresinski S. Strongyloidiasis in solid organ transplantation. OBM Transplant. 2018;2:035.

[36] Rosen A, Ison MG. Screening of living organ donors for endemic infections: understanding the challenges and benefits of enhanced screening. Transpl Infect Dis. 2017;19:e12633. 10.1111/tid.12633 27862705

[37] Camargo JF, Simkins J, Anjan S, Guerra G, Vianna R, Salama S, et al. Implementation of a *Strongyloides* screening strategy in solid organ transplant donors and recipients. Clin Transplant. 2019;33:e13497. 10.1111/ctr.13497 30773692

[38] Kottkamp AC, Filardo TD, Holzman RS, Aguero-Rosenfeld M, Neumann HJ, Mehta SA. Prevalence of strongyloidiasis among cardiothoracic organ transplant candidates in a non-endemic region: a single-center experience with universal screening. Transpl Infect Dis. 2021;23:e13614. 10.1111/tid.13614 33844416

[39] Roxby AC, Gottlieb GS, Limaye AP. Strongyloidiasis in transplant patients. Clin Infect Dis. 2009;49:1411–23. 10.1086/630201 19807271 PMC2913967

[40] Bisoffi Z, Buonfrate D, Sequi M, Mejia R, Cimino RO, Krolewiecki AJ, et al. Diagnostic accuracy of five serologic tests for *Strongyloides stercoralis* infection. PLoS Negl Trop Dis. 2014;8:e2640. 10.1371/journal.pntd.0002640 24427320 PMC3890421

[41] Kalantari N, Chehrazi M, Ghaffari S, Gorgani-Firouzjaee T. Serological assays for the diagnosis of *Strongyloides stercoralis* infection: a systematic review and meta-analysis of diagnostic test accuracy. Trans R Soc Trop Med Hyg. 2020;114:459–69. 10.1093/trstmh/trz135 32052848

[42] Buonfrate D, Formenti F, Perandin F, Bisoffi Z. Novel approaches to the diagnosis of *Strongyloides stercoralis* infection. Clin Microbiol Infect. 2015;21:543–52. 10.1016/j.cmi.2015.04.001 25887711

